# Prospective Validation of a Prognostic Model for Respiratory Syncytial Virus Bronchiolitis in Late Preterm Infants: A Multicenter Birth Cohort Study

**DOI:** 10.1371/journal.pone.0059161

**Published:** 2013-03-12

**Authors:** Maarten O. Blanken, Hendrik Koffijberg, Elisabeth E. Nibbelke, Maroeska M. Rovers, Louis Bont, on behalf of the Dutch RSV Neonatal Network

**Affiliations:** 1 Department Pediatric Immunology and Infectious Diseases, University Medical Center, Utrecht, Utrecht, The Netherlands; 2 Department Julius Center for Health Sciences and Primary Care, University Medical Center Utrecht, Utrecht, The Netherlands; 3 Departments of Epidemiology, Biostatistics & HTA, and operating rooms, Radboud University Nijmegen Medical Center, Nijmegen, The Netherlands; University of Liverpool, United Kingdom

## Abstract

**Objectives:**

This study aimed to update and validate a prediction rule for respiratory syncytial virus (RSV) hospitalization in preterm infants 33–35 weeks gestational age (WGA).

**Study Design:**

The RISK study consisted of 2 multicenter prospective birth cohorts in 41 hospitals. Risk factors were assessed at birth among healthy preterm infants 33–35 WGA. All hospitalizations for respiratory tract infection were screened for proven RSV infection by immunofluorescence or polymerase chain reaction. Multivariate logistic regression analysis was used to update an existing prediction model in the derivation cohort (n = 1,227). In the validation cohort (n = 1,194), predicted versus actual RSV hospitalization rates were compared to determine validity of the model.

**Results:**

RSV hospitalization risk in both cohorts was comparable (5.7% versus 4.9%). In the derivation cohort, a prediction rule to determine probability of RSV hospitalization was developed using 4 predictors: family atopy (OR 1.9; 95%CI, 1.1–3.2), birth period (OR 2.6; 1.6–4.2), breastfeeding (OR 1.7; 1.0–2.7) and siblings or daycare attendance (OR 4.7; 1.7–13.1). The model showed good discrimination (c-statistic 0.703; 0.64–0.76, 0.702 after bootstrapping). External validation showed good discrimination and calibration (c-statistic 0.678; 0.61–0.74).

**Conclusions:**

Our prospectively validated prediction rule identifies infants at increased RSV hospitalization risk, who may benefit from targeted preventive interventions. This prediction rule can facilitate country-specific, cost-effective use of RSV prophylaxis in late preterm infants.

## Introduction

Respiratory syncytial virus (RSV) bronchiolitis is one of the most common causes of infant hospitalization during the winter season and is associated with a large burden of disease and high costs.[1]–[5] Hospitalization for RSV lower respiratory tract infection in Europe and the United States is estimated to be 1–3% of all infants aged less than 13 months. Important risk groups for RSV bronchiolitis are infants with prematurity with or without chronic lung disease, congenital heart disease, Down syndrome and immunodeficiencies.[6]–[9] Although risk groups for RSV bronchiolitis have been identified, the precise incidence of hospitalization for RSV bronchiolitis in these patient populations is generally not known. There is no effective therapy for RSV infection, so treatment is mainly symptomatic.[10] Due to the increased risk most high risk groups receive RSV immunoprophylaxis to prevent RSV infection. Palivizumab, a humanized immunoglobin monoclonal antibody, specific for RSV, has been proven effective and safe for preterm infants with gestational age ≤35 weeks, infants with bronchopulmonary dysplasia and infants with congenital heart disease.[11], [12] Efficacy of 55% of RSV prophylaxis has been demonstrated for late preterm infants 33–35 weeks gestational age (WGA). Subgroup analysis showed 80% efficacy of RSV prophylaxis in 32–35 WGA preterm infants.[12] In many countries RSV immunoprophylaxis is not used in late preterm infants 33–35 WGA because of high costs.[13] Within health care, limited budgets force the need to selectively apply high cost treatments to a proportion of infants identified as having increased risk for severe disease. Costs may be reduced by targeting RSV immunoprophylaxis to 33–35 WGA late preterm infants with additional risk factors.[14] Several environmental and clinical risk factors have been described which compound the risk for severe RSV disease. Presence of siblings, daycare attendance, month of birth and protective factors like breastfeeding have been described as independent risk factors for severe disease due to RSV infection.[15]–[21] In a recent paper it was emphasized that validated prediction rules are required to improve the care of our patients with infectious diseases.[22] Two prediction rules for late preterm infants 33–35 WGA have been published but these have not yet been validated prospectively.[23], [24] To develop a practical and accurate prediction model for the Netherlands the prediction rule previously developed by Simoes et al. may have inferior performance in countries, such as the Netherlands, in which most children visit day care facilities.[24] We therefore aimed to update and validate a RSV prediction rule for 33–35 WGA late preterm infants using 2 prospective birth cohorts.[24]

## Methods

### Study design

RISK is an ongoing study prospectively performed in late preterm infants born at 32 weeks and 1 day to 35 weeks and 6 days weeks gestational age (referred to as 33–35 WGA) in 41 hospitals of the RSV Neonatal Network in the Netherlands. Between June 2008 and January 2011 infants were included in hospitals located across the Netherlands. The study population consisted of newborn infants born at 33–35 WGA from 1 university hospital and 40 regional hospitals. Infants with gross abnormalities or Down syndrome, and those who received palivizumab for any reason were excluded. The study consists of 2 subsequent birth cohorts: a derivation cohort and a validation cohort.

### Ethics statement

The study was reviewed and approved by the Institutional Review Board of the University Medical Center Utrecht and subsequently approved by Institutional Review Boards of all participating hospitals. All parents provided written informed consent for screening of hospital records. The study was conducted in compliance with the Declaration of Helsinki and the standards of Good Clinical Practice.

### Data collection

At birth, a questionnaire containing questions on family history of wheeze, asthma and hay fever, smoking during pregnancy and in the household, the number of siblings and their age, parental education level, potential breastfeeding, potential day-care attendance, household pets and pregnancy details was filled out by parents. Clinical data on the mode of delivery, gestational age, respiratory support, birth weight, Apgar score and delivery details were derived from patient charts. The following 7 variables from the prediction rule previously developed by Simoes et al. were noted: ‘birth within 10 weeks of the start of the season,’ ‘birth weight,’ ‘breast-feeding ≤2 months,’ ‘number of siblings ≥2 years of age,’ ‘number of family members with atopy,’ ‘male sex,’ and ‘number of family members with wheeze’[24]. Breast-feeding was defined as either exclusive breastfeeding or mixed with formula feeding. Atopy was defined as the presence of asthma, eczema or hay fever. At one year of age, parents were contacted by telephone to determine whether hospitalization for respiratory disease had occurred. If any data were missing from questionnaires completed by the parents/legal guardians or from the clinical records, the respective physician was contacted for information, which ensured that all baseline data were assembled. If the parents could not be reached by telephone, the hospital and general practitioner were contacted for updated information. If no valid telephone number was available, an e-mail or letter was sent to the parents.

### Outcome definition

When parents reported hospitalization for respiratory disease during the first year of life, we analysed the medical hospital record for RSV hospitalization, including routine virology results. The main study endpoint, hospitalization for RSV bronchiolitis was defined as hospitalization for lower respiratory tract infection with proven RSV infection determined by routine practice laboratory testing in the participating hospitals, i.e. either by rapid RSV immunofluorescence test or polymerase chain reaction.

### Statistical analysis

Sample size calculation: According to a generally accepted rule of thumb that at least 10 cases are required per variable in the prediction rule. For a 7-variable model we calculated a priori, a sample size of 70 infants hospitalized for RSV bronchiolitis.[24] With an estimated incidence of 4%, the projected sample size of the derivation cohort was 1,750. To validate a 4-variable prediction rule, the estimated sample size of the validation cohort was 1,000.

### Derivation and validation of the prediction rule

We assessed the test performance of the clinical prediction rule to identify infants at high risk for hospitalization with RSV bronchiolitis. To evaluate the models' calibration, the Hosmer-Lemeshow statistic was used in which observations are grouped based on deciles of predicted probability and compared with the observed risk of RSV bronchiolitis in the derivation and validation cohort. This was graphically assessed with a calibration plot and tested with the Hosmer-Lemeshow statistic, where a non-significant test indicated good model fit.[25], [26] Discrimination is the ability of the rule to distinguish between infants hospitalized from those not hospitalized for RSV bronchiolitis, and will be quantified with the Area Under the Receiver Operating Characteristic curve (AUROC). An AUROC area ranges from 0.5 (no discrimination.) to 1.0 (perfect discrimination).

We anticipated that the prediction rule previously developed by Simoes et al. may have inferior performance in countries, such as the Netherlands, in which most children visit day care facilities. Therefore we planned to update the model. Multivariable logistic regression was used to update the independent contribution of each of the variables to the discrimination of the model. The updated model was reduced by excluding variables from the model with univariate p-values >0.15, using the log likelihood ratio test. The AUROC was used to determine whether the variables provided added predictive value beyond the existent prediction rule.[27] Other, additional variables with a univariate p-value of <0.15 not included in the original prediction rule were added to increase the discrimination and reliability of the prediction rule. Subsequently, the model shrinkage was applied in the derivation dataset using bootstrapping, to adjust the model's estimated regression coefficients in order to reduce overfitting.[25], [28] We repeated the modelling process in 1,000 bootstrap samples. For each individual infant the risk score was calculated using the bootstrap-corrected coefficients of the updated prediction rule. The value of each risk factor was multiplied by its coefficient and the sum of all resulting values and the model intercept, i.e. the linear predictor, was calculated. The results of the validation were examined primarily by classification tables and by calculating the AUROC. To make the model easy to use in a clinical setting we calculated a point score.

The updated prediction rule was externally validated in a new cohort of infants. The two cohorts were derived by making a non-randomized split according to birth date.[29]

We defined our derivation cohort as all infants born between June 2008 and September 2009, and our validation cohort as all infants born between September 2009 and January 2011. We calculated performance of the rule as sensitivity, specificity, positive likelihood ratio and negative likelihood ratio. Statistical analysis was performed by using SPSS 15.0. (SPSS Inc, Chicago, Ill).

## Results

### Patient characteristics

**Figure 1:**
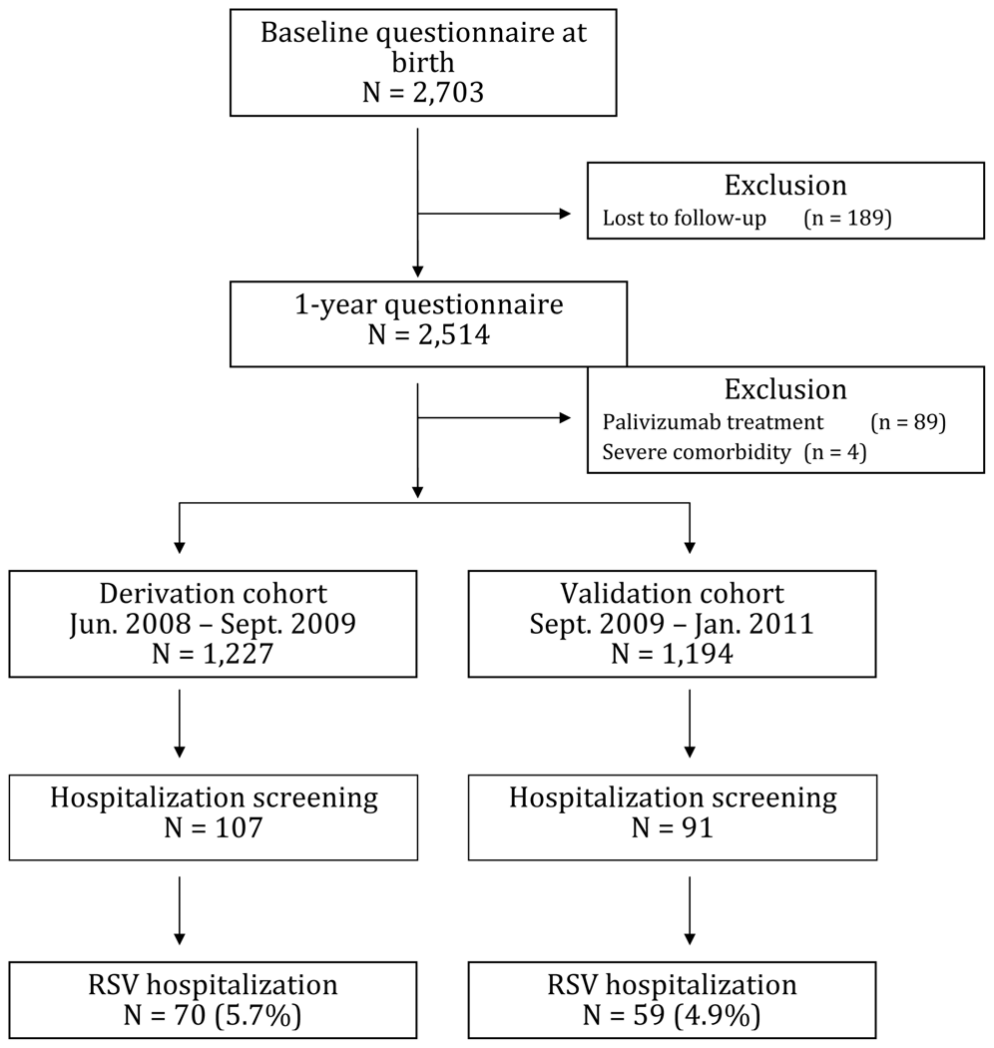
Patient flowchart derivation and validation cohort.

In total, 2,703 infants born in the 41 participating hospitals were included (figure 1, table 1); 186 infants (7%) were lost to follow-up after a year. Three infants died of RSV-unrelated causes. Of the 2,514 included infants, 198 parents reported hospitalization for respiratory tract symptoms during the first year of life and these were verified through hospital medical records. For these 198 hospitalizations, tests for RSV were positive in 129 instances (5.1%) and negative in another 41 (1.6%). Testing for RSV was not performed in 28 cases.

**Table 1:** Distribution of Baseline Patient Characteristics in the Derivation and Validation Cohort (Number(percentage)).

	Derivation cohort (n = 1,227)	Validation cohort (n = 1,194)
Male gender	676 (55.1%)	659 (55.2%)
Gestational age (wk)	34	34
32	115 (9.4%)	124 (10.4%)
33	296 (24.1%)	240 (20.1%)
34	371 (30.2%)	429 (35.9%)
35	445 (36.3%)	401 (33.6%)
Birth Weight (g) (Mean(SD))	2214 (452)	2225 (427)
Multiple pregnancy	426 (34.7%)	422 (35.3%)
Caesarean section	409 (33.3%)	436 (36.5%)
Continuous positive airway pressure	166 (13.5%)	217 (18.2%)
Mechanical ventilation*	46 (3.7%)	35 (2.9%)
Born Aug 14 th to Dec 1 st	324 (26.4%)	496 (41.5%)
Breastfeeding** less than 2months or not ^#^	416 (33.9%)	376 (31.5%)
Presence of siblings	504 (41.1%)	463 (38.8%)
Atopy in 1^st^ degree family member	642 (52.3%)	729 (61.1%)
Fur bearing pets	571 (46.5%)	548 (45.9%)
Maternal smoking during pregancy	164 (13.4%)	136 (11.4%)
Subject daycare attendance ^#^	730 (59.5%)	714 (59.8%)
Number of house hold residents (Median (95%CI))	3 (2–4)	3 (2–4)
Siblings or subject daycare attendance	959 (78.2%)	918 (76.9%)

* No infants developed BPD ** either exclusive breastfeeding or mixed with formula feeding # predicted by parents at birth.

### Derivation of the prediction rule

Table 2 shows the distribution of potential predictors of RSV bronchiolitis. In the derivation cohort we updated a previously published prediction rule.[24] Of the seven predictors in this original model the following four variables ‘birth within 10 weeks of the start of the season,’ ‘breast-feeding ≤2 months’, ‘number of siblings ≥2 years of age’, ‘number of family members with atopy’, contributed significantly. Updating the model by adjusting the four original variables to increase discrimination and by stepwise backward selection in the derivation cohort resulted in the final 4-variable model including ‘born Aug 14 th to Dec 1 st’, ‘presence of siblings or day care attendance’, ‘atopy in a 1^st^ degree family member’ and ‘breast-feeding ≤2 months’. The AUROC of this updated model was 0.703 (95% CI 0.64–0.76) before bootstrapping and 0.702 (0.64–0.76) afterwards (Table 3). We used point values generated from the five times multiplied and rounded regression coefficients to develop a score. We entered the scores of each patient in a logistic regression model to generate the individual predicted probability of RSV hospitalization. For scores ≥16 mean predicted probabilities were 10.0% (95% CI 7.0–14.2%) versus 3.5% in scores <16.

**Table 2:** Distribution of potential predictors across cases and non-cases in the derivation and validation cohort.

	Derivation cohort (n = 1,227)		Validation cohort (n = 1,194)	
Characteristic (Number (%))	RSV hospitalization (n = 70)	Controls (n = 1,157)	RSV hospitalization (n = 59)	Controls (n = 1,135)
Born Aug 14 th to Dec 1 st	32 (45.7%)	292 (25.2%)	35 (59.3%)	461 (40.6%)
Gestational age (weeks) (Median (95%CI))	34 (32−35)	34 (32−35)	34(32−35)	34 (32−35)
Birth weight, gr (Mean (SD))	2216 (483)	2214 (450)	2215 (395)	2200 (428)
Breast fed* ≤ 2 months or not^#^	32 (45.7%)	384 (33.2%)	20 (33.9%)	356 (31.4%)
Presence of siblings	46 (65.7%)	458 (39.6)	33 (55.9%)	430 (37.9%)
Atopy in 1^st^ degree family member	46 (65.7%)	596 (51.5%)	41 (69.5%)	688 (60.6%)
Male gender	39 (55.7%)	637 (55.1%)	29 (49.2%)	630 (55.5%)
Fur bearing pets	27 (38.6%)	544 (47.0%)	22 (37.3%)	526 (46.3%)
Maternal smoking during pregancy	11 (15.7%)	153 (13.2%)	9 (15.3%)	127 (11.2%)
Subject daycare attendance^#^	47 (67.1%)	683 (59.0%)	41 (70.7%)	673 (59.4%)
Number of residents	3.1 (0.84)	2.8 (0.80)	3.0 (0.80)	3.0 (0.80)
Siblings or subject daycare attendance	66 (94.3%)	893 (77.2%)	55 (93.2%)	863 (76.0%)
Multiple birth	25 (35.7%)	401 (34.7%)	14 (23.7%)	408 (36.1%)

* either exclusive breastfeeding or mixed with formula feeding # predicted by parents at birth.

**Table 3:** Results of the multivariable logistic regression analyses in the derivation cohort (n = 1227) and the performance of the model in the validation cohort (n = 1194): predictors for RSV hospitalization after bootstrapping.

Characteristics	RISK model			RISK point score
	Regression coefficient	Odds ratio (95% CI)	p-value	
*Born Aug 14 th to Dec 1 st*	0.96	2.6 (1.6−4.2)	<0.001	5
*Presence of siblings or subject daycare attendance* ^#^	1.65	4.7 (1.7−13.1)	0.003	8
*Breast fed* * *2months or not* ^#^	0.51	1.7 (1.0−2.7)	0.04	3
*Atopy in 1^st^ degree family member*	0.67	1.9 (1.1−3.2)	0.01	3
				
*Intercept*	−4.20			
AUROC (95%CI) derivation cohort		0.702 (0.64−0.76)		
AUROC (95%CI) validation cohort		0.678 (0.61−0.74)		

* either exclusive breastfeeding or mixed with formula feeding # predicted by parents at birth.

### Validation of the prediction rule

In our independent validation sample, the updated prediction rule demonstrated satisfactory discrimination (AUROC, 0.678; 95% CI 0.61–0.74) (Table 3). In the calibration plot, the intercept was 0.0, the slope was 1.0, indicating good calibration. The Hosmer-Lemeshow test resulted in a p-value of 0.26, and the average absolute difference in predicted and calibrated probabilities was 0.008. We calculated sensitivity, specificity and diagnostic likelihood ratios for each score defined as high-risk categories (Table 4). Using a threshold score ≥16 we observed that 27 infants (positive predictive value 10%) were hospitalized for RSV bronchiolitis in the validation cohort. We calculated the following other characteristics of the RISK prediction rule: negative predictive value of 96%, sensitivity of 46% (95% CI 34–58%), a specificity of 79% (95% CI 76–81%), a positive likelihood ratio of 2.1 (95% CI 1.6–2.9) and a negative likelihood ratio of 0.7 (95% CI 0.5–0.9).

**Table 4:** Operating Characteristics for Each Threshold of the RISK model in the validation cohort (n = 1194).

	RISK score			
	≥ 8	≥11	≥16	≥19
True positive	56 (4.7%)	53 (4.4%)	27 (2.3%)	8 (0.7%)
False positive	957 (80%)	745 (62%)	243 (20%)	62 (5.0%)
True negative	178 (15%)	390 (33%)	892 (75%)	1073 (90%)
False negative	3 (0.2%)	6 (0.5%)	32 (2.6%)	51 (5.0%)
Sensitivity	0.95	0.90	0.46	0.14
Specificity	0.16	0.34	0.79	0.95
Positive likelihood ratio	1.1	1.4	2.1	2.5
Negative likelihood ratio	0.3	0.3	0.7	0.9

## Discussion

We showed that the overall RSV hospitalization risk was 5.1% in this population of healthy late preterm infants 33–35 WGA. As far as we are aware, this is the first prospective validation study for RSV hospitalization in late preterm infants. The sample size was large enough for both updating and validating the updated prediction rule. The 4-variable prediction rule can be used to further target preventive interventions at those infants who have the highest risk for hospitalization caused by RSV infection.

Two previous studies described prediction rules for RSV hospitalization in late preterm infants.[23], [24] The group of Figueras-Aloy developed a 7-variable prediction rule for RSV hospitalization in a group of late preterms born between 33–35 weeks of gestation. This model was retrospectively validated in French, Italian and Danish cohort studies or case-control studies.[30]–[33] We updated the Spanish prediction rule aiming to produce a model which is both valid and practical in clinical use. The predictors in our prediction rule are also in agreement with a Canadian prediction model.[31] This model was retrospectively validated in the case-control study used to develop the Spanish prediction rule.[23] Although the Canadian study has not been prospectively validated, this study is used for targeted prophylaxis in Canada. The performance of the RISK prediction model is remarkably similar to the actual impact of the Canadian model as it targets 22% of the late preterm cohort which is comparable to the performance of the prediction rule used in Canada which targets 18% of late preterms of 33–35 WGA.[31]

The major strengths of our study include: that data from 2 large prospective cohorts were collected allowing further validation of an existing RSV prediction rule, the retrieval of complete baseline data, and palivizumab was used by less than 5% in our study population because it is not reimbursed. The majority of infants who received palivizumab in our study population had either a congenital anomaly or chronic lung disease. Some potential limitations included the following. First, an underestimation of RSV hospitalization may have occurred, because not all infants hospitalized for respiratory tract infections were routinely tested. Underestimation of the risk of RSV hospitalization is unlikely to have affected the AUROC of the prediction rule, but would result in an underestimation of the positive predictive value. Second, of all infants with a score <16, 3.5% will be hospitalized for RSV bronchiolitis while not classified as high risk. Third, 6.1% of parents could not be contacted after 1 year despite attempts to obtain contact details via the hospital, general practitioner or a web-based search and this could be a potential selection bias. Since the vast majority of parents were contacted we believe this does not significantly jeopardize the conclusions of this study. Fourth, this study does not answer the on-going question of cost-effectiveness of RSV immunoprophylaxis in late preterm infants.[13], [14], [34]–[39] Conflicting reports on this matter have recently been published.[36], [40]–[42] However, applying the RISK prediction rule will certainly improve cost-effectiveness of RSV prophylaxis. Five, because there is no gold standard for RSV prediction we were unable to assess the criterion validity of the RISK prediction model. Content, construct and face validity were accounted for because our analyses covered all relevant RSV risk factors and the outcome of our model is based on laboratory confirmed RSV hospitalizations. Since we externally validated the prediction model in a prospective and independent second cohort we believe the model was sufficiently validated.

The RISK prediction model incorporates four simple clinical variables which combined can be used for risk stratification in the birth period among late preterm infants. The RISK model provides an important foundation for targeted prevention for those infants most at risk for severe RSV disease. With the RISK prediction rule a high risk group can be identified with a hospitalization risk >10% which is comparable to the hospitalization risk in preterm infants <32 weeks gestational age and other high risk groups.[6], [7] If a risk score of 16 is applied, then infants with a risk score exceeding this threshold comprise 22% of all preterm infants 33–35 weeks gestational age. By targeting only 22% of this large birth cohort of late preterm infants for prophylaxis, the potential impact of our model is not dissimilar to the Canadian findings.[31] Future research should focus on the confirmation of the impact of the RISK prediction rule during implementation in clinical guidelines.

## Conclusion

The risk of hospitalization for RSV bronchiolitis in late preterms is 5.1%. The RISK prediction rule is a simple clinical rule identifying a subgroup of 33–35 WGA late preterm infants with increased risk of hospitalization for RSV bronchiolitis. Implementation of the RISK prediction rule will further improve cost-effectiveness of RSV prophylaxis.
